# Transcriptomic and Metabolomic Analyses Reveal the Response Mechanism of *Ophiopogon japonicus* to Waterlogging Stress

**DOI:** 10.3390/biology13030197

**Published:** 2024-03-20

**Authors:** Tingting Cheng, Xia Zhou, Juan Lin, Xianjian Zhou, Hongsu Wang, Tiezhu Chen

**Affiliations:** 1 Sichuan Academy of Chinese Medicine Sciences, Chengdu 610041, China; 2018055932@nwsuaf.edu.cn (T.C.); zhouxia.sc@163.com (X.Z.); lj20231030@126.com (J.L.); 18200572680@163.com (X.Z.); hongsu_wang@163.com (H.W.); 2 Sichuan Provincial Key Laboratory of Quality and Innovation Research of Chinese Materia Medica, Chengdu 610041, China

**Keywords:** *Ophiopogon japonicus*, transcriptome, metabolome, waterlogging, responding mechanism

## Abstract

**Simple Summary:**

*Ophiopogon japonicus* is susceptible to flooding because it grows in river alluvial dams. In order to avoid the growth and development of *Ophiopogon japonicus* from being affected by flooding, this project analyzed the changes in the genetic information of *Ophiopogon japonicus* under flooding stress and identified genetic data that can enhance the flooding tolerance of *Ophiopogon japonicus*. These experimental results lay the foundation for breeding flood-tolerant *Ophiopogon japonicus*.

**Abstract:**

*Ophiopogon japonicus*, a plant that thrives in river alluvial dams, often faces waterlogging stress due to sustained rainfall and flood seasons, which significantly impacts its growth and development. Currently, the mechanisms of waterlogging tolerance in *Ophiopogon japonicus* are still unclear. This study analyzed the transcriptome and metabolome data for *Ophiopogon japonicus* in the Sichuan region (referred to as CMD) under varying degrees of waterlogging stress: mild, moderate, and severe. The results indicate that the group exposed to flooding stress exhibited a higher number of differentially expressed genes (DEGs) compared to the control group. Notably, most DEGs were downregulated and primarily enriched in phenylpropanoid biosynthesis, starch and sucrose metabolism, and plant hormone signal transduction pathways. A total of 5151 differentially accumulated metabolites (DAMs) were identified, with significantly upregulated DAMs annotated to two clusters, namely flavonoids such as apiin, pelargonin, and others. Furthermore, our study revealed significant upregulation in the expression of C2H2 (C2H2 zinc finger proteins) and AP2/ERF-ERF (the subfamily ERF proteins of APETALA2/ethylene-responsive element binding factors) transcription factors in CMD under flooding stress, suggesting their critical roles in enabling CMD to adapt to these conditions. In conclusion, this research provides insights into the intricate molecular mechanisms underlying CMD’s response to flooding stress and reports valuable genetic data for the development of transgenic plants with improved waterlogging tolerance.

## 1. Introduction

*Ophiopogon japonicus* (L. f.) Ker-Gawl (abbreviated as MD), which belongs to the family Liliaceae, is a medicinal plant distributed in East Asia. Its dried tuberous roots are used in traditional Chinese medicine [1]. MD cultivation is well-suited for the first and second terraces of river alluvial dams due to the flat terrain and the presence of primarily fresh alluvial soil with a moderate clay and sand content, meeting the ideal growth conditions for MD. These river terraces also offer a network of self-flowing irrigation channels, ensuring an adequate water supply for MD growth. However, the downside is their susceptibility to waterlogging and flooding, imposing waterlogging stress on MD. To mitigate the detrimental effects of waterlogging stress, agricultural practices such as ditching and draining, plowing and tilling, and increased application of organic fertilizers have been implemented in production. However, these measures are associated with higher labor costs [2]. Thus, the most effective approach and pivotal breeding objective remain the development of MD varieties with enhanced flood tolerance to simplify cultivation practices and reduce costs. Presently, most research on waterlogging tolerance in MD has predominantly centered on the physiological aspects of resistance, leaving a substantial gap in our understanding of the underlying molecular mechanisms.

Transcriptomic and metabolomic analyses are widely used in the field of plant molecular research. Transcriptomics primarily focuses on analyzing gene expression in plants across various developmental stages and under different environmental conditions to elucidate the mechanisms governing plant growth, development, and adaptation to their surroundings. Comparatively, metabolomics is used to study alterations in the composition and content of metabolites within specific temporal and spatial contexts to establish connections between metabolites and physiological changes in plants. These powerful analytical tools have been instrumental in investigating diverse aspects of plant biology including the molecular mechanisms underlying plant growth, development, and the synthesis of active compounds [3,4,5,6], responses to pests and pathogens [7,8,9,10], and the tolerance mechanisms associated with abiotic stresses [11,12,13,14]. However, there is limited transcriptomic and metabolomic research on MD under conditions of waterlogging stress.

MD primarily originates from regions in Sichuan and Zhejiang [15]. Sichuan MD (abbreviated as CMD) represents a cultivated variety derived from the wild MD population in the Sichuan area [16]. It possesses several advantages including a shorter planting duration, higher yield, and lower cost, which contribute to its dominant market presence and make it the predominant MD species [17]. Therefore, CMD was selected for transcriptome and metabolome analyses in this study, and the critical pathways of CMD adaptation to waterlogging stress were identified by comparing the transcript levels of CMD genes and metabolite accumulation under different degrees of waterlogging stress. These research results reveal new genetic information on MD and the potential for improving the quality, varieties, and waterlogging tolerance of MD.

## 2. Materials and Methods

### 2.1. Plant Material and Growing Conditions

CMD specimens were obtained from Sichuan Province, China (31°30′ N, 104°95′ E and 31°32′ N, 104°89′ E) and botanically identified as *Ophiopogon japonicus* (L. f.) Ker-Gawl by the researcher Tiezhu Chen. Tiller seedlings were cultivated in plastic pots in the greenhouse facilities of the Sichuan Academy of Chinese Medicine Sciences (30°37′ N, 104°40′ E). During the growth and development of the CMD germplasm resources, watering was controlled, and other cultivation management practices were kept consistent. After an acclimatization period of 28 days, the seedlings were subjected to controlled watering conditions and randomly allocated into four groups: control (BF-1), light waterlogging (CF-1), moderate waterlogging (MF-1), and heavy waterlogging (SF-1), each comprising three pots. The BF-1 group received standard watering, while the CF-1 group was exposed to waterlogging conditions up to one-third of the roots, the MF-1 group was submerged up to two-thirds of the roots, and the SF-1 group was inundated up to the rhizome junction. The pots had a caliber of 24 cm, a base diameter of 19.5 cm, and a height of 26.5 cm. The substrate was formulated with an organic matter content of 1.80 g/kg, total N 2.50 g/kg, total P 1.50 g/kg, pH 6.8, and a maximum water holding capacity of the substrate of 25.87%. After 21 days of incubation under these conditions, the roots were collected, washed with distilled water, immediately frozen in liquid nitrogen, and stored at −80 °C for subsequent transcriptome and metabolome analyses [18].

### 2.2. Transcriptome Analysis

RNA isolation and library construction were performed according to the manufacturer’s protocols with the RNeasy Plant Mini Kit (Qiagen, Germany) and NEBNext^®^Ultra^™^ RNA Library Preparation Kit for Illumina^®^ (NEB, Ipswich, MA, USA). The primer sequences (5′-3′): AGATCGGAAGAGCGTCGTGTAGGGAAAGAGTGT; AGATCGGAAGAGCACACGTCTGAACTCCAGTCAC were used. A cDNA library was constructed using CMD samples and sequenced in PE150 mode utilizing the Illumina NovaSeq6000 sequencing platform [19,20]. The raw sequencing data underwent a series of preprocessing steps that involved the removal of reads containing adapters, reads containing poly-N sequences, and low-quality reads. This preprocessing resulted in the generation of high-quality clean data. Transcriptome assembly was performed using Trinity [21], with default parameters except for the “min_kmer_cov” parameter, which was set to 2. Gene functions were annotated by referencing several databases including NR (NCBI non-redundant protein sequences) [22], Pfam (Protein family) [23], KOG/COG/eggNOG (Clusters of Orthologous Groups of proteins) [24], Swiss-Prot (a manually annotated and reviewed protein sequence database) [25], KEGG (Kyoto Encyclopedia of Genes and Genomes) [26], and GO (Gene Ontology) [27]. Intergroup DEGs were analyzed for BF-1, CF-1, MF-1, and SF-1 using the DESeq R package (1.10.1) [28], and DEGs were functionally clustered via the KEGG pathway and GO [29].

### 2.3. Metabolite Identification

The total metabolites of the CMD samples were extracted by initially weighing 50 mg of the sample, followed by the addition of 1000 μL of an extraction solution containing an internal standard (methanol: acetonitrile: water, 2:2:1, *v/v*, with 2-chloro-L-phenylalanine (20 mg/L). The mixture was vortexed for 30 s and then subjected to mechanical disruption for 10 min using a grinder at 45 Hz, followed by a 10-min ultrasonication step in an ice-water bath. Afterward, the sample was allowed to stand at 20 °C for 1 h and subsequently centrifuged at 4 °C and 12,000 rpm for 15 min. From the resulting supernatant, 500 μL was collected and vacuum-dried. Following this, 160 μL of an extraction solution (acetonitrile:water, 1:1, *v/v*) was added, vortexed for 30 s, sonicated in an ice-water bath for 10 min, and centrifuged at 4 °C and 12,000 rpm for 15 min. The analyzed sample was processed using an LC-MS system (Aquity I-Class PLUS Ultra High-Performance Liquid Tandem Waters Xevo G2-XS QTOF High-Resolution Mass Spectrometer) with separation achieved using an Aquity UPLC HSS T3 column (1.8 μm, 2.1 × 100 mm) acquired from Waters. Metabolites were detected in both cationic and anionic modes on a Waters Xevo X2-XS QTof high-performance liquid spectrometer using the MSe mode, and the data were obtained using MassLynx V4.2 software (Waters), which enabled the acquisition of primary and secondary mass spectrometry data simultaneously. In each data acquisition cycle, dual-channel data acquisition was conducted for both low and high collision energies, where the low collision energy was set at 2 V, and the high collision energy ranged from 10 to 40 V. The parameters for the ESI ion source were configured as follows: capillary voltage of 2500 V for positive ion mode and −2000 V for negative ion mode, cone-well voltage of 30 V, ion source temperature of 100 °C, desolventization gas temperature of 500 °C, blowback gas flow rate of 50 L/h, desolventization gas flow rate of 800 L/h, and a mass-to-charge ratio (*m/z*) acquisition range of 50–1200. The raw data acquired using MassLynx V4.2 were processed for peak extraction and peak alignment using Progenesis QI software (4.0) [30]. The reproducibility of the samples was evaluated by principal component analysis (PCA). Metabolites were annotated utilizing the Kyoto Encyclopedia of Genes and Genomes (KEGG), Human Metabolome Database (HMDB), and lipid profiles [31]. Based on the grouping information, the degree of differences was computed and compared, and the significance levels (*p*-values) for each compound were determined using a *t*-test. The OPLSDA model was constructed using the R language software (3.6.1) package ropls [32], and the model’s reliability was confirmed by 200 alignment tests. Multiple cross-validations were conducted to calculate the VIP value of the model. Differentially expressed metabolites were identified using a combination of criteria including fold change (FC > 1), *p*-value (*p*-value < 0.05), and VIP value (VIP > 1). Statistical analysis of the metabolome data was performed using the BMKCloud platform (Biotechnology Co. Ltd., Beijing, China).

### 2.4. Real-Time Quantitative PCR Validation

Six genes were randomly selected for qRT-PCR. The specific primers were designed using Primer Premier 5.0 software; details are shown in Table 1. The qRT-PCR was performed in a 20 μL reaction mixture consisting of 10 μL of 2× ChamQ SYBR Color qPCR Master Mix, 2 μL each of the upstream and downstream primers, 4 μL of template cDNA, and ddH2O was added to reach a total volume of 20 μL. The reaction conditions were as follows: initial denaturation at 95 °C for 30 s, followed by 40 cycles of 15 s at 95 °C, and 30 s at 60 °C. The relative mRNA expression levels were calculated using the 2^−ΔΔCt^ method, and all experiments were conducted in triplicate.

### 2.5. Statistical Analysis

Excel 2019 software was used for statistical analysis. Bar graphs are shown using the mean ± SD of three independent experiments.

## 3. Results

### 3.1. Analysis of Transcriptome Results

**Figure 1:**
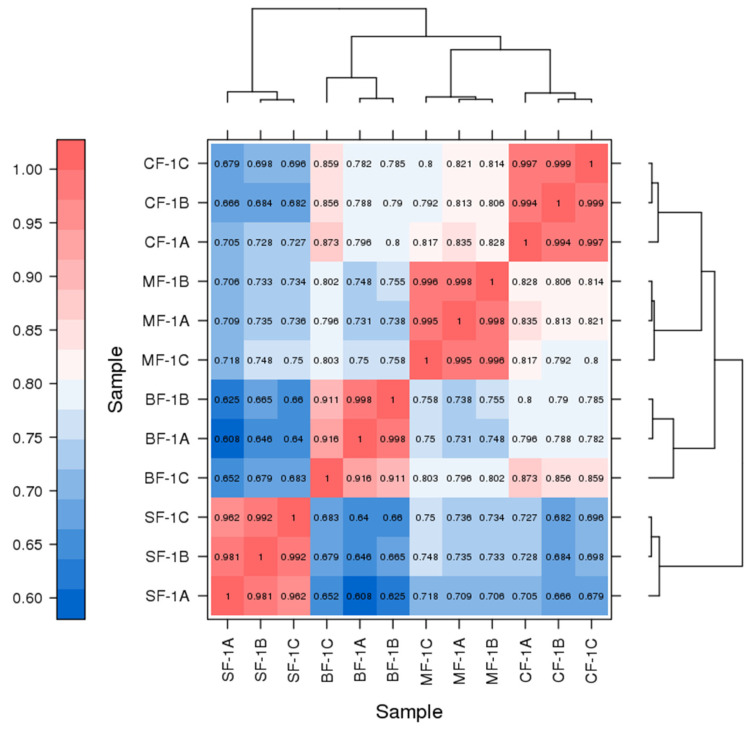
Correlation between the CMD samples obtained following waterlogging treatment. ABC indicates different replicates within the same group. The color of the box represents the degree of correlation, with dark red and dark blue representing the highest and the lowest degree of correlation.

**Figure 2:**
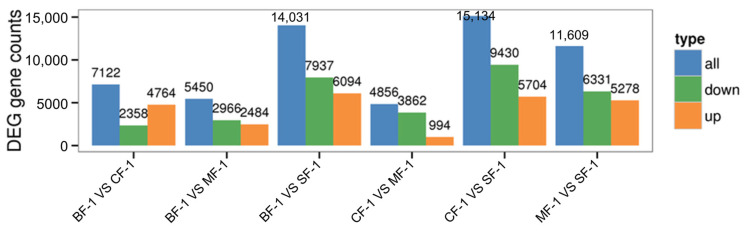
Total differentially expressed genes.

Three root samples were collected from each of the following groups: blanks, mild waterlogging (BF-1), moderate waterlogging (CF-1), and heavy waterlogging (SF-1) for transcriptome sequencing, yielding a total of 12 qualified libraries, from which clean reads were obtained by eliminating low-quality reads, resulting in approximately 76.25 Gb of clean data. The clean data of each sample exceeded 5.73 Gb, with Q30 bases accounting for over 91.21%. A total of 53,704 unigenes were obtained after assembly. The assessment of the gene expression level correlations among samples is important for verifying the reproducibility of biological experiments, evaluating the reliability of differentially expressed genes (DEGs), and identifying any outliers. In this study, Pearson’s correlation coefficient (PC) analysis was used to assess the correlations and reproducibility among the samples. As shown in Figure 1, duplicate samples exhibited significant clustering, indicating consistency among biological replicates and reliable sequencing results. Differential gene screening, based on the criteria of |Fold Change| ≥ 2 and FDR < 0.01, showed that the number of DEGs exceeded 11,000 between SF-1 and the other three groups, with a notable predominance of downregulated genes (Figure 2). In contrast, the number of DEGs between CF-1 and MF-1 was minimal, signifying minor gene expression variations between mild and moderate waterlogging conditions, aligning with the findings presented in Figure 1.

### 3.2. Differential Gene Function Analysis

**Figure 3:**
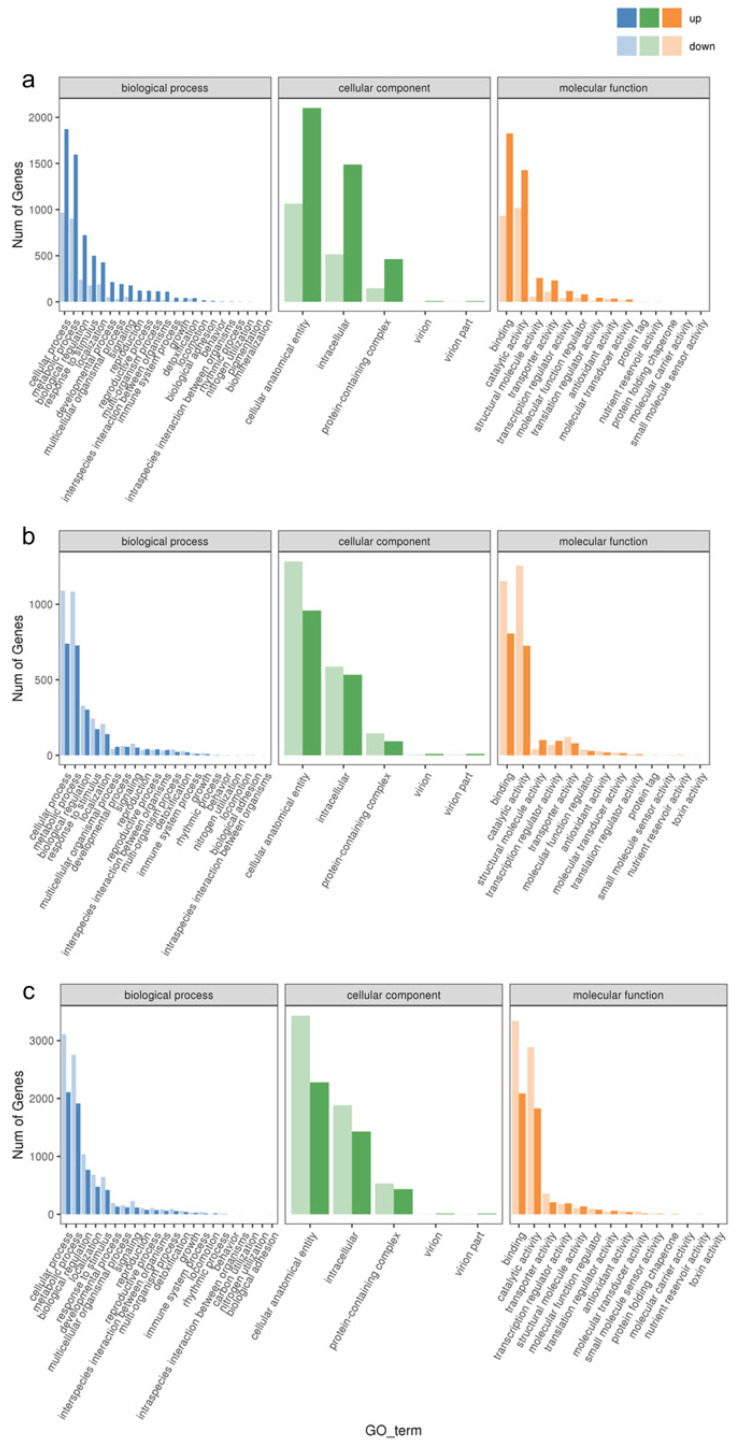
Enrichment of GO entries for DEGs. (**a**) Enrichment of DEGs for BF-1 vs. CF-1. (**b**) Enrichment of DEGs for BF-1 vs. MF-1. (**c**) Enrichment of DEGs for BF-1 vs. SF-1.

In order to verify the biological functions of the differentially expressed genes in response to the waterlogging stress treatments, we annotated these genes into the Gene Ontology (GO) Database and classified them into three main categories: biological process, cellular component, and molecular function. As shown in Figure 3, the top two enriched GO entries remained consistent across various levels of waterlogging stress. In the biological process category, the differential genes were predominantly enriched in cellular processes and metabolic processes. Likewise, in the cellular component category, these genes exhibited enrichment in cellular anatomical entities and intracellular components. In the molecular function category, the DEGs were primarily associated with binding and catalytic activity. Notably, there were variations in the number of upregulated and downregulated DEGs among the different treatment groups. Specifically, CF-1 demonstrated a higher number of upregulated DEGs compared to downregulated DEGs, while the opposite trend was observed for MF-1 and SF-1, where downregulated DEGs exceeded the upregulated DEGs in number.

**Figure 4:**
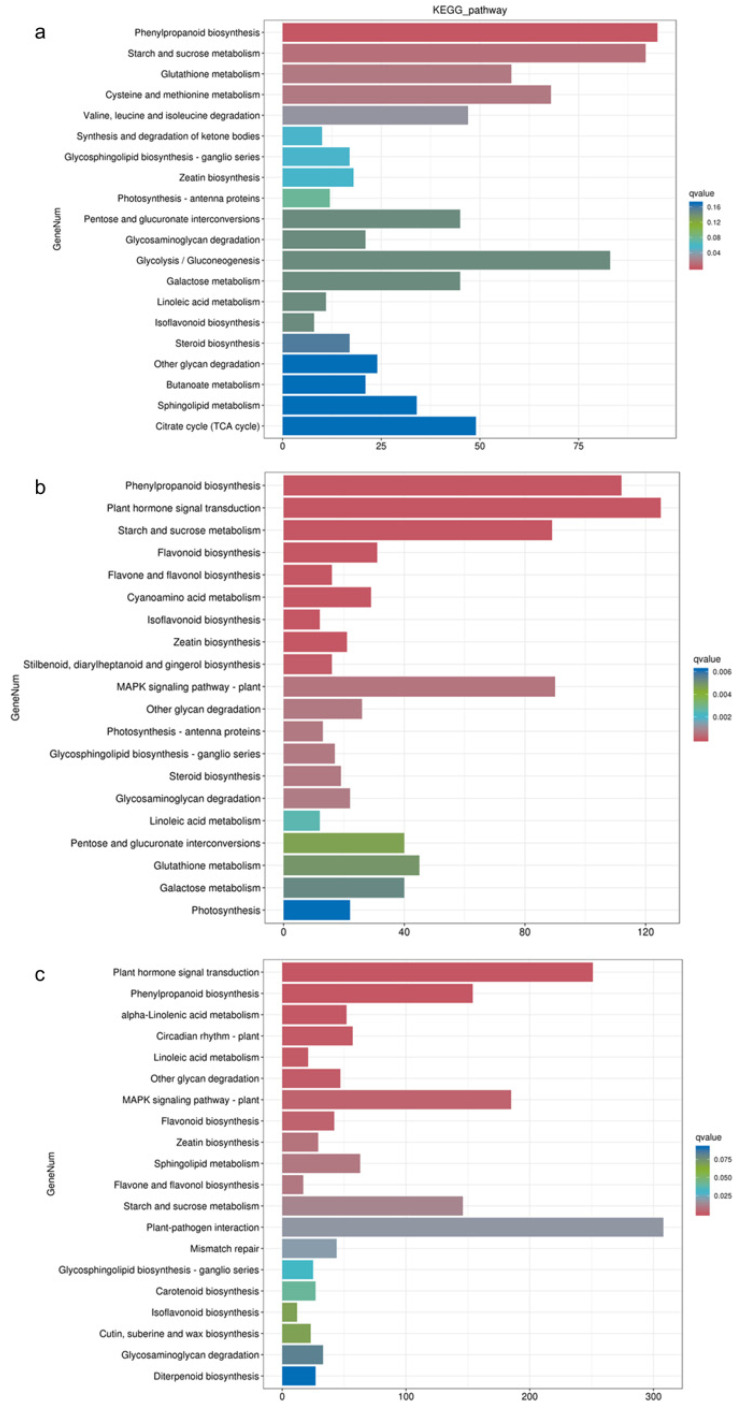
KEGG enrichment of DEGs. (**a**) Enrichment of DEGs for BF-1 vs. CF-1. (**b**) Enrichment of DEGs for BF-1 vs. MF-1. (**c**) Enrichment of DEGs in BF-1 vs. SF-1.

The KEGG enrichment analysis revealed distinct pathway enrichments in response to different waterlogging treatments (Figure 4). In the CF-1 group, the DEGs were significantly enriched in pathways related to phenylpropanoid biosynthesis, starch and sucrose metabolism, and cysteine and methionine metabolism. For the MF-1 group, the DEGs exhibited significant enrichment in phenylpropanoid biosynthesis, plant hormone signal transduction, and the starch and sucrose metabolism pathways. In the SF-1 group, the differential genes were significantly enriched in pathways related to plant hormone signal transduction, phenylpropanoid biosynthesis, and the MAPK signaling pathway-plant pathway. Overall, it can be deduced that phenylpropanoid biosynthesis plays a pivotal role in the response of CMD to waterlogging stress across all treatments, and as the severity of waterlogging increased, there was a notable transition in the primary response pathway. Specifically, the shift was observed from starch and sucrose metabolism in CF-1 and MF-1 to plant hormone signal transduction in MF-1 and SF-1.

### 3.3. Metabolomic Analysis

**Figure 5:**
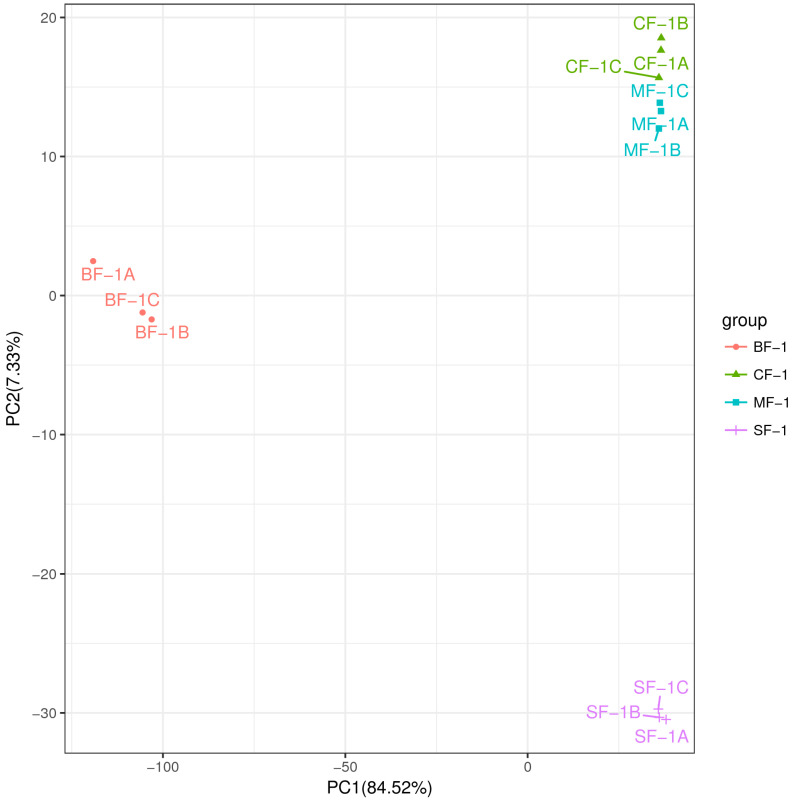
Principal component analysis (PCA) between the samples.

**Figure 6:**
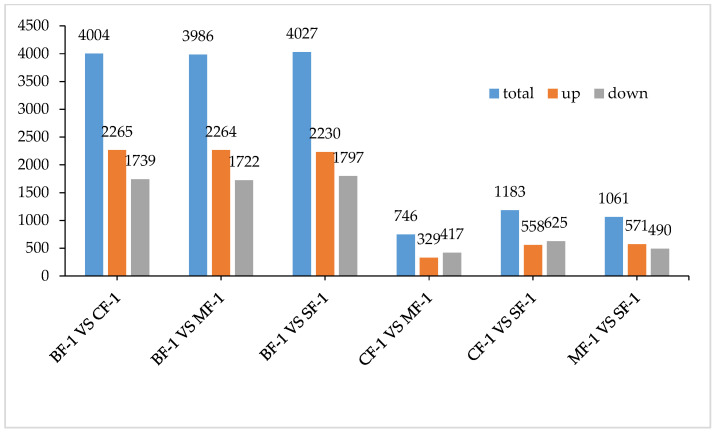
Total differential metabolites.

To investigate the inherent material differences within CMD in response to waterlogging stress, LC-QTOF-MS metabolomics was performed to identify differential metabolites. Principal component analysis (PCA) revealed a high level of data consistency among replicate samples within each group (Figure 5), demonstrating clear separation of metabolites in CMD subjected to varying degrees of waterlogging treatments. Metabolite identification in this study was based on data detected in both positive and negative ion modes, resulting in the annotation of a total of 5151 peaks. As illustrated in Figure 6, the flooded group displayed approximately 4000 differential metabolites (DAMs) compared to the blank group, with approximately 2265 upregulated and 1700 downregulated DAMs. Remarkably, the influence of waterlogging severity on DAMs was relatively minor. Specifically, the number of upregulated DAMs decreased proportionally from mild to moderate waterlogging but increased in the moderate waterlogging group compared to the heavy waterlogging group. In essence, the variations in DAMs between different waterlogging levels were small. However, a significant difference was observed between the flooded and blank groups in the number of DAMs.

### 3.4. The Material Basis of CMD Response to Waterlogging Stress

**Figure 7:**
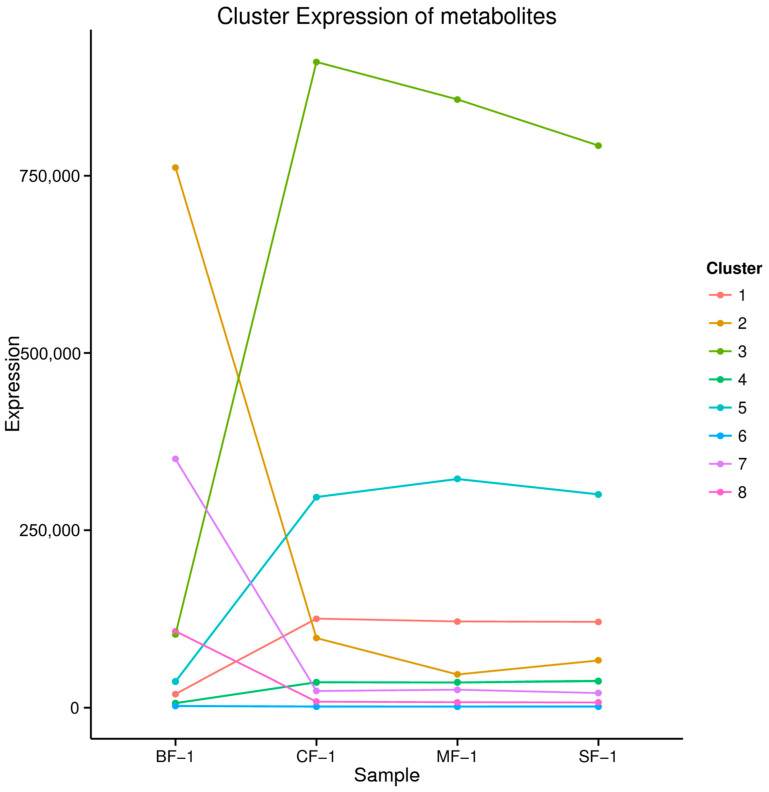
Kmeans_cluster of differential metabolites.

The 5151 DAMs were categorized into eight clusters based on their abundance and variance (Figure 7). Notably, the contents of metabolites in cluster 1, cluster 3, cluster 4, and cluster 5 increased after waterlogging, while the contents of metabolites in clusters 2, 7, and 8 decreased, and that of cluster 6 remained relatively stable. Cluster 3, encompassing four metabolites, and cluster 5, comprising 21 metabolites, displayed significantly higher levels of metabolites such as homoharringtonine, apiin, pelargonin, and chelidonic acid in the flooded group compared to the blank group (Table 2). Collectively, our findings suggest that these compounds may constitute the material basis for the adaptation of CMD following submersion due to waterlogging stress.

### 3.5. Key Pathways of CMD in Response to Waterlogging Stresses

**Figure 8:**
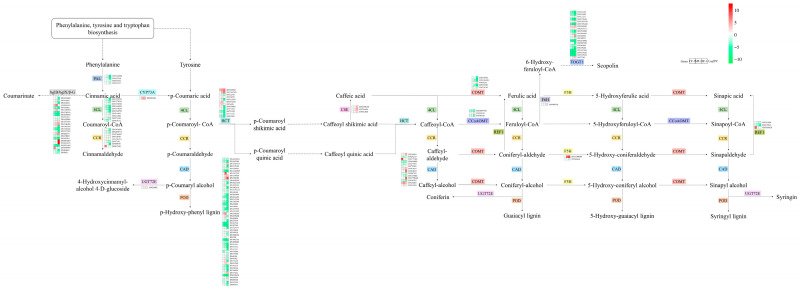
Differences in the phenylpropanoid biosynthesis pathway in CMD under different levels of waterlogging. Heat maps representing the relative transcript levels of the enzymes. PAL: phenylalanine ammonia-lyase; 4CL: 4-coumarate-CoA ligase; CCR: cinnamoyl-CoA reductase; bglB/bglX/β-G: beta-glucosidase; CYP73A: trans-cinnamate 4-monooxygenase; CAD: cinnamyl-alcohol dehydrogenase; UGT72E: coniferyl-alcohol glucosyltransferase; POD: peroxidase; HCT: shikimate O-hydroxycinnamoyltransferase; CSE: caffeoylshikimate esterase; COMT: caffeic acid 3-O-methyltransferase/acetylserotonin O-methyltransferase; CCoAOMT: caffeoyl-CoA O-methyltransferase; REF1: coniferyl-aldehyde dehydrogenase; F5H: ferulate-5-hydroxylase; F6H: feruloyl-CoA 6-hydroxylase; TOGT1: scopoletin glucosyltransferase.

Phenylalanine and tyrosine play vital roles in the waterlogging tolerance of CMD. After exposure to waterlogging, the phenylpropanoid biosynthesis pathway of CMD exhibited a response involving 20 key enzymes and 36 metabolites (Figure 8). Notably, the expression levels of enzymes such as PAL, 4CL, and CCR decreased proportionally with the severity of waterlogging. CMD’s strategy for coping with waterlogging stress was found to primarily involve reducing phenylalanine and tyrosine metabolism while increasing their accumulation.

### 3.6. Key TFs of CMD in Response to Waterlogging Stresses

**Figure 9:**
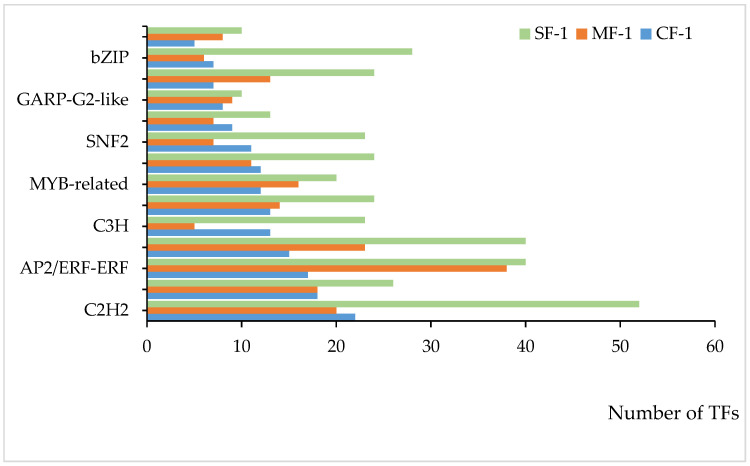
TFs shared among the top 20 in each of the three waterlogging groups.

Multiple transcription factors (TFs) played a significant role in regulating the tolerance of CMD to waterlogging. As shown in Figure 9, 14 of the top 20 ranked differential TFs between the treatment and blank groups were common across all three waterlogging treatment groups, representing a 70% overlap. Among these, the top five TFs included C2H2, AP2/ERF-ERF, bHLH, and NAC, with four of them being shared. Together, these four TFs accounted for 34.6% to 43.4% of the total among the top 20 TFs, highlighting their pivotal role in regulating CMD’s waterlogging tolerance.

### 3.7. Validation of qRT-PCR

**Figure 10:**
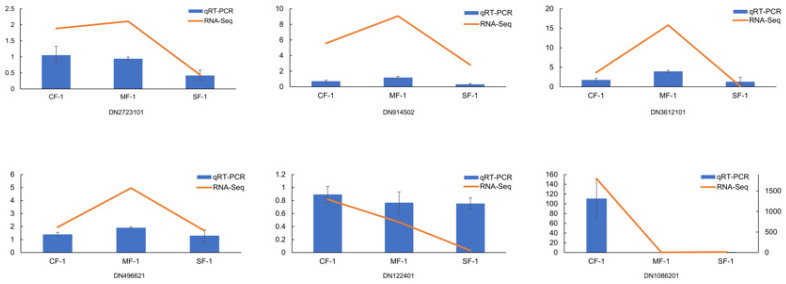
Expression verification of 6 key genes involved in phenylalanine and tyrosine metabolism in CMD.

To validate the accuracy of the DEGs, six key DEGs (DN2723101, DN914502, DN3612101, DN496621, DN122401, and DN1086201) involved in phenylalanine and tyrosine metabolism were selected for qRT-PCR validation. The results demonstrated that the expression trend of the six selected genes in each sample was consistent with the RNA-Seq expression levels, which highly confirmed the reliability and credibility of the RNA-Seq results (Figure 10).

## 4. Discussion

The analysis of transcriptomic and metabolomic variations in CMD exposed to varying degrees of waterlogging stress provides valuable insights into the intricate metabolic adjustments CMD undergoes to adapt to such stress conditions. In the subsequent sections, we comprehensively discuss these gene expression products and metabolites to shed light on these vital aspects of the response of CMD to waterlogging stress.

### 4.1. Impact of Waterlogging on the CMD Transcriptome and Metabolome

Metabolic regulation is an important mechanism enabling plants to sustain their physiological functions during waterlogging [33,34,35,36]. Plant gene transcription and product metabolism actively engage in multiple metabolic pathways to adapt to such stressors. In the context of waterlogging stress, different plant varieties can exhibit significant impacts on metabolic pathways and gene transcription levels. For instance, flood-tolerant onions demonstrated a higher prevalence of downregulated genes [37], while cotton displayed a robust response involving antioxidant enzyme genes and transcription factor genes after 20 days of waterlogging [14]. Similarly, waterlogging stress induced notable changes in gene expression and metabolites in CMD. Interestingly, under mild waterlogging stress, CMD exhibited a greater number of upregulated DEGs compared to downregulated DEGs. In contrast, CMD subjected to moderate and severe waterlogging stress displayed a higher number of downregulated DEGs than upregulated DEGs. Overall, across all inundation groups, the number of upregulated DEGs exceeded the downregulated DEGs, with minimal variation observed between the groups, suggesting that waterlogging stress significantly influences gene expression and metabolite content in CMD, but their responses differ based on the stress severity. While the degree of stress has a substantial impact on gene expression, it has a comparatively lesser effect on the number of metabolites.

### 4.2. Significantly Adjusted Biosynthesis Responses in Waterlogged CMD

**Figure 11:**
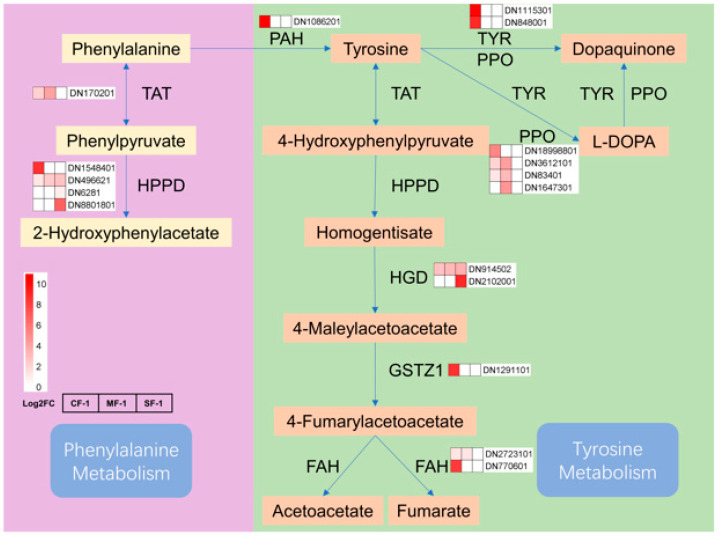
Phenylalanine and tyrosine metabolism in waterlogged CMD. The relative transcript levels of the enzyme are represented using a heat map. TAT: tyrosine aminotransferase; HPPD: 4-hydroxyphenylpyruvate dioxygenase; PAH: phenylalanine-4-hydroxylase; TYR: tyrosinase; PPO: polyphenol oxidase; HGD: homogentisate 1,2-dioxygenase; GSTZ1: maleylacetoacetate isomerase; FAH: fumarylacetoacetase.

The tricarboxylic acid (TCA) cycle in mitochondria constitutes the respiratory metabolism of plant cells and serves as a vital pathway for providing energy to various organelles, thereby sustaining essential physiological functions [38]. Previous research has indicated that TCA cycle metabolites such as succinate, α-ketoglutarate, and fumarate are reduced in drought-tolerant maize following water stress [39]. Interestingly, after subjecting CMD to waterlogging stress, most enzymes in the phenylpropanoid biosynthesis pathway displayed negative feedback regulation, with their levels positively correlated with the severity of waterlogging, which was directly proportional to the degree of waterlogging. The reduction in the gene transcription of metabolic enzymes increased the accumulation of phenylalanine and tyrosine, indirectly boosting the gene transcription activity of enzymes in the phenylalanine and tyrosine metabolism pathways (Figure 11). The elevated levels of fumarate induced changes in the tricarboxylic acid cycle pathway, subsequently impacting the metabolism of sugars, fatty acids, and amino acids. These findings suggest that the regulation of primary metabolism, particularly energy metabolism, may represent one of the strategies employed by CMD to adapt to waterlogging stress. In addition, proteins involved in carbon metabolic pathways such as glycolysis are also important material bases in response to waterlogging stress [40,41,42], but need to be further explored and validated in CMD.

Secondary metabolites play a pivotal role in regulating various physiological activities of plants, enabling them to adapt to diverse abiotic stresses [43,44,45,46]. For instance, *Brassica napus*, subjected to drowning stress, exhibited a significant enrichment of different genes in metabolic pathways including the biosynthesis of secondary metabolites and flavonoid biosynthesis [33]. In soybeans, the content of flavonoids showed a proportional increase with drought stress severity [47]. In the case of *Syntrichia caninervis*, after exposure to drought conditions, the differentially expressed transcripts were significantly enriched in pathways such as phenylpropanoid biosynthesis, which positively correlated with indicators such as absolute water content [48]. Similarly, after CMD underwent waterlogging stress, the levels of phenylpropanoid metabolism and flavonoid metabolism, initiated by phenylalanine and tyrosine, were significantly impacted, resulting in the increased accumulation of downstream metabolites and their derivatives including pelargonin, apiin, schaftoside 4′-O-glucoside (Table 1). These observations suggest that alterations in the accumulation of secondary metabolites, particularly flavonoids, represent another mechanism through which CMD adapts to waterlogging stress.

In addition, the mechanism of plant response to waterlogging stress is also closely related to plant species and duration of flooding. For example, onion (*Allium cepa* L.) showed significant changes in the phenylalanine metabolic pathway, pathogenesis-related proteins, and energy production after 72 h of waterlogging treatment [37]. Cotton (*Gossypium hirsutum* L.) showed a significant increase in several metabolites including sinapyl alcohol, adenosine, and galactaric acid after 10–20 days of waterlogging [14]. The mechanisms by which CMD responds to waterlogging stress are different from the literature, but there are similarities. After 21 days of waterlogging, the metabolite contents of apii, pelargonin, and other metabolites were increased in CMD, which was different from the response mechanism in cotton. The phenylalanine metabolic pathway also showed a downregulation in gene transcription like onion, so it suggests that the phenylalanine metabolic pathway is one of the important pathways in plant response to waterlogging stress.

### 4.3. Differentially Expressed TF Responses in Waterlogged CMD

**Figure 12:**
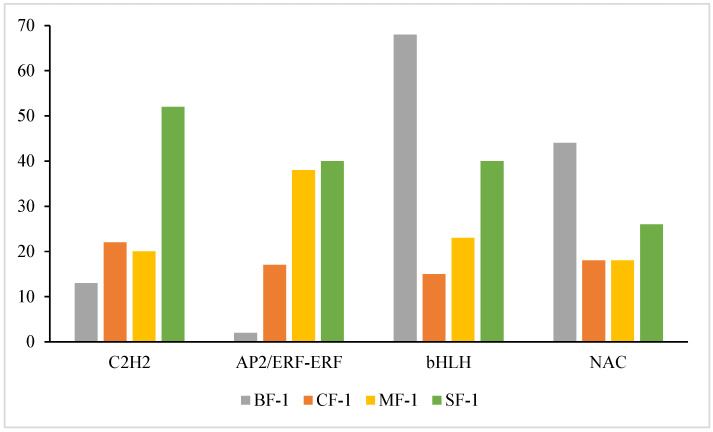
Amounts of transcription factors C2H2, AP2/ERF-ERF, bHLH, and NAC in CMD.

TFs hold significant importance in the responses of plants to abiotic stressors [49,50,51,52]. Previous studies have established that members of the C2H2, AP2/ERF-ERF, bHLH, and NAC TF families play crucial roles in the abiotic stress responses of plants and their ability to tolerate waterlogging stress [53,54,55,56,57,58]. For instance, C2H2 and NAC TFs contribute to maintaining iron homeostasis in waterlogged rice, a region known for Fe^2+^ toxicity [59]. C2H2 is involved in tissue and organ development in cucumbers and plays a role in responding to various abiotic stresses such as drought, cold, and salt [60]. Additionally, waterlogging stress has been associated with the upregulation of AP2/ERF-ERF and the downregulation of bHLH in kiwifruit [61,62]. In this study, differential expression of these TFs was also observed (Figure 12). The TFs C2H2, AP2/ERF-ERF, bHLH, and NAC in CMD correlated positively with the severity of waterlogging stress as a whole. Notably, the expression of C2H2 and AP2/ERF-ERF was higher than that of the control, while the expression of bHLH and NAC was lower than that of the control, aligning with the existing literature, suggesting that C2H2, AP2/ERF-ERF, bHLH, and NAC are key transcription factors regulating CMD genes under waterlogging stress, especially C2H2 and AP2/ERF-ERF. C2H2 can regulate the effects of waterlogging stress by activating the ABA pathway [63]. ERF-Ⅶ is a subgroup of AP2/ERF-ERF that mediates hypoxia signaling in plants and interacts with calcium-dependent protein kinases to enhance plant hypoxia sensing [64,65].

## 5. Conclusions

In this study, we exposed CMD to mild, moderate, and severe waterlogging stress treatments. Comprehensive transcriptomic and metabolomic analyses revealed that the phenylpropanoid biosynthesis pathway in CMD exhibited negative feedback regulation in response to waterlogging stress, leading to an increased accumulation of phenylalanine and tyrosine, which impacted the phenylalanine metabolism, tyrosine metabolism, phenylpropanoid metabolism, and flavonoid metabolism pathways. Additionally, the expression of transcription factors C2H2 and AP2/ERF-ERF was significantly upregulated, suggesting their pivotal role in the adaptation of CMD to waterlogging stress. These findings contribute valuable insights into the gene regulatory mechanisms involved in enhancing flood tolerance for breeding flood-tolerant CMD varieties.

## Figures and Tables

**Table 1:** List of qRT-PCR primers in this study.

Gene Name	Primer Sequence (5’-3’)
actin-F	ATTCCCAAGGCAGCCACAA
actin-R	ATACAGACACCCAGCCTCCTTTA
DN2723101-F	CGTTCCTCAACAACTCCCAA
DN2723101-R	GAAGAAAAGAAGTCAGTGTAATCCC
DN914502-F	TTCACATGTATGCTGCTAACAAGTC
DN914502-R	CAGGTAAGTCTACTGCAAAACGAAA
DN3612101-F	CATAGGCACAACCACGGCA
DN3612101-R	AATTTTCACCGTCCCAGCC
DN496621-F	TAGGCCGACAATATTTCTAGAGATC
DN496621-R	AATCCTCCACATCCGCCTT
DN122401-F	ACCACCATCTCATCAAACTCCTCA
DN122401-R	ATACAGCGTGAAGGTCAAGTCCC
DN1086201-F	CAAACCTAGAGTGCATGACCATAAT
DN1086201-R	GAGGGATTTTATCTCCATAGCGA

**Table 2:** Types and relative contents of differential metabolites in clusters 3 and 5.

Cluster	Compounds	Molecular Formula	Molecular Weight (g/mol)	HMDB_taxonomy
cluster3	Homoharringtonine	C29H39NO9	545.62	Cephalotaxus alkaloids
cluster3	Apiin	C26H28O14	564.49	Naphthalenes
cluster3	All-trans-phytofluene	C40H62	542.92	Prenol lipids
cluster3	Pelargonidin 3-O-beta-D-sambubioside	C26H29O14	565.50	--
cluster5	Copal-8-ol diphosphate	C20H38O8P2	468.50	--
cluster5	Ganoderenic acid A	C30H42O7	514.65	Prenol lipids
cluster5	Solamargine	C45H73NO15	868.06	Azaspirodecane derivatives
cluster5	OA-6129 A	C20H31N3O7S	457.55	--
cluster5	O-Phospho-L-homoserine	C4H10NO6P	199.02	Carboxylic acids and derivatives
cluster5	(3S,2′S)-4-Ketomyxol 2′-alpha-L-fucoside	C46H64O8	745.00	--
cluster5	Bacoside A3	C47H76O18	929.10	--
cluster5	Schaftoside 4′-O-glucoside	C32H38O19	726.60	Flavonoids
cluster5	Pelargonin	C27H31O15	595.526	Flavonoids
cluster5	Muzanzagenin	C27H38O5	442.60	Steroids and steroid derivatives
cluster5	Amaranthin	C30H34N2O19	726.59	Betalains
cluster5	DL-4-Hydroxy-2-ketoglutarate	C5H6O6	162.10	--
cluster5	PG(16:0/0:0)	C22H45O9P	484.60	--
cluster5	D-Maltose	C12H22O11	342.30	Organooxygen compounds
cluster5	3.alpha.-Mannobiose	C12H22O11	342.30	--
cluster5	Chelidonic acid	C7H4O6	184.10	Pyrans
cluster5	ω-Pentadecalactone	C15H28O2	240.38	--
cluster5	Gypenoside XVII	C48H82O18	947.20	Prenol lipids
cluster5	PG(20:2(11Z,14Z)/16:1(9Z))	C42H77O10P	773.00	--
cluster5	Dioctyl phthalate	C24H38O4	390.60	Benzene and substituted derivatives
cluster5	DG(18:3(9Z,12Z,15Z)/18:3(9Z,12Z,15Z)/0:0)	C39H64O5	612.90	Fatty Acyls

## Data Availability

The data presented in this study are available on request from the corresponding author. The data are not publicly available due to our research is on going.
